# An LC-MS/MS Method for Analysis of Vitamin D Metabolites and C3 Epimers in Mice Serum: Oral Supplementation Compared to UV Irradiation

**DOI:** 10.3390/molecules26175182

**Published:** 2021-08-26

**Authors:** Amir Sohail, Asma Al Menhali, Soleiman Hisaindee, Iltaf Shah

**Affiliations:** 1 Department of Chemistry, College of Science, United Arab Emirates University (UAEU), Al Ain 15551, United Arab Emirates; 201770097@uaeu.ac.ae (A.S.); soleiman.hisaindee@uaeu.ac.ae (S.H.); 2 Department of Biology, College of Sciences, United Arab Emirates University (UAEU), Al Ain 15551, United Arab Emirates

**Keywords:** C3 epimer, 25OHD, vitamin D, LC-MS/MS, mice serum, oral supplementation

## Abstract

Introduction: The most common forms of vitamin D in human and mouse serum are vitamin D3 and vitamin D2 and their metabolites. The aim of this study is to determine whether diet and sunlight directly affect the circulating concentrations of vitamin D metabolites in a mouse model. We investigated the serum concentrations of eight vitamin D metabolites—vitamin D (vitamin D3 + vitamin D2), 25OHD (25OHD3 + 25OHD2), 1α25(OH)_2_D (1α25(OH)_2_D2, and 1α25(OH)_2_D3)—including their epimer, 3-epi-25OHD (3-epi-25OHD3 and 3-epi-25OHD2), and a bile acid precursor 7alpha-hydroxy-4-cholesten-3-one (7αC4), which is known to cause interference in liquid chromatography-tandem mass spectrometry (LC-MS/MS) analysis. Method: The LC-MS/MS method was validated according to FDA-US guidelines. The validated method was used for the analysis of mouse serum samples. Forty blood samples from mice were collected and divided into three groups. The first group, the DDD mice, were fed a vitamin D-deficient diet (25 IU VD3/kg of diet) and kept in the dark; the second group, the SDD mice, were maintained on a standard-vitamin D diet (1000 IU VD3) and kept in the dark; and the third group, SDL, were fed a standard-vitamin D diet (1000 IU VD3) but kept on a normal light/dark cycle. LC-MS/MS was used for the efficient separation and quantitation of all the analytes. Results: The validated method showed good linearity and specificity. The intraday and interday precision were both <16%, and the accuracy across the assay range was within 100 ± 15%. The recoveries ranged between 75 and 95%. The stability results showed that vitamin D metabolites are not very stable when exposed to continuous freeze–thaw cycles; the variations in concentrations of vitamin D metabolites ranged between 15 and 60%. The overlapping peaks of vitamin D, its epimers, and its isobar (7αC4) were resolved using chromatographic separation. There were significant differences in the concentrations of all metabolites of vitamin D between the DDD and SDL mice. Between the groups SDD (control) and SDL, a significant difference in the concentrations of 3-epi-25OHD was noted, where C3 epimer was about 30% higher in SDL group while no significant differences were noted in the concentrations of vitamin D, 25OHD, 1α25(OH)_2_D, and 7αC4 between SDD and SDL group. Conclusions: A validated method, combined with a simple extraction technique, for the sensitive LC-MS/MS determination of vitamin D metabolites is described here. The method can eliminate the interferences in LC-MS/MS analysis caused by the overlapping epimer and isobar due to them having the same molecular weights as 25OHD. The validated method was applied to mouse serum samples. It was concluded that a standard-vitamin D diet causes an increase in the proportion of all the vitamin D metabolites and C3 epimers and isobar, while UV light has no pronounced effect on the concentrations of the majority of the vitamin D metabolites except 3-epi-25OHD. Further studies are required to confirm this observation in humans and to investigate the biochemical pathways related to vitamin D’s metabolites and their epimers.

## 1. Introduction

Bioactive vitamin D is the most common steroidal hormone and plays a very prominent role in many biological processes in humans and animal models such as cardiovascular [1,2], nervous [3], skeletal [4,5], and reproductive systems [6]; calcium and phosphorus homeostasis [7,8,9]; hypertension [10,11]; chronic kidney disease (CKD) [12,13]; psoriasis [14]; cancer [15]; and autoimmune diseases [16,17]. The collective term vitamin D includes both vitamin D3 and vitamin D2. 

**Figure 1:**
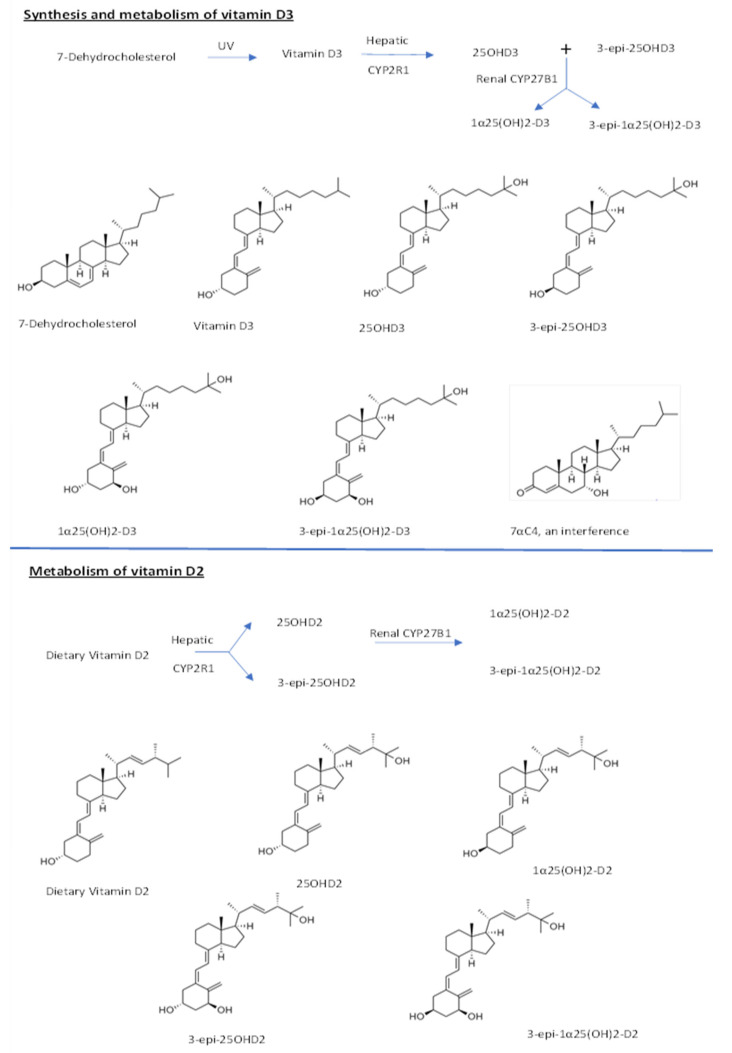
Metabolic pathways for vitamins D2, D3, and chemical structures of metabolites.

Vitamin D3 is obtained from food and/or by the conversion of 7-dehydrocholesterol under the action of UVB light on the skin. [18,19]. Vitamin D3 is then transported to the liver, where it is hydroxylated at the 25 positions by the enzyme cytochrome p450 (CYP2R1), to produce 25-hydroxyvitamin-D3 (25OHD3) and its epimer 3-epi-25OHD3. In the kidney, renal 1-α-hydroxylase enzyme (CYP27B1) further hydroxylate 25OHD3 into the 1α25(OH)_2_D3 and its epimer 3-epi 1α25(OH)_2_D3 [20]. Vitamin D2, on the other hand, is obtained only from dietary sources [21,22] and is metabolized in a similar way to produce 25-hydroxyvitamin-D2 (25OHD2) and 1α25(OH)_2_D2 and their epimers as depicted in Figure 1.

1α25(OH)_2_D3 is considered to be the most effective vitamin form of vitamin D active. Its influence (genomic or non-genomic) is partly mediated by its high binding affinity toward vitamin D receptors (VDRs), the nuclear vitamin D receptor (nVDR), and membrane vitamin D receptor (mVDR) [23]. Epimers of vitamin D also play important physiological roles. Recent rat studies have shown that epimers of vitamin D are involved in bone mineral density (BMI) and overall growth [24]. It is also known that C3 epimers are elevated in the sera of mice supplemented with dietary vitamin D3 [25]. It is also reported that 3-epi-1α25(OH)_2_D3 can initiate gene transcription through the VDR, as can 1α25(OH)_2_D3 [26]. 

In humans, neonates and infants have higher concentrations (upto 60%) of vitamin D epimers until the age of one year [27,28]. The proportion of 3-epi-25OHD relative to 25OHD3 varies between 0 and 45%. Supplementation with vitamin D3 has been shown to increase the concentration of 3-epi-25OHD3 in a linear fashion. In another study, it was indicated that high levels of 3-epi-25OHD3 are present in some pregnant women and fetuses, and the high C3 epimer concentrations in infancy are most likely to be due to early embryogenesis [20,23]. 

Given the importance of vitamin D in the body, many analytical methods have been developed for the analysis of vitamin D metabolites in animal and human models. By far, the most efficient is the LC-MS/MS method, as it does not suffer from the flaws associated with immunoassay techniques [29]. Its advantages include the short run time, the use of an internal standard that allows to evaluate the instrumental and matrix-related effects [30], and its efficiency in separating the interfering co-eluting epimers and isobars [31,32,33]. 7αC4 is a bile acid precursor with the same molecular weight as 25OHD3 and its C3 epimer, and it is also known to interfere with vitamin D LC-MS/MS analysis [28,34,35,36,37]. 

In this study, we aimed to develop and validate an improved LC-MS/MS method in mouse serum with an analytical advantage to better quantify vitamin D metabolites in mouse serum as compared to previous research. This research further aimed to investigate the effect of different vitamin D dietary and UV light exposure conditions on the level of vitamin D metabolites, epimers, and isobar in mice population. For efficient separation and quantitation, we modified a recently published LC-MS/MS method for vitamin D analysis [30]. The metabolic pathways for vitamins D2, D3, and the chemical structures of all the metabolites, epimers, and isobar are given in the Figure 1 below.

## 2. Results

The validation results are summarized in Table 1. The reported linear range for all the analytes was 0.5–150 ng/mL, except for 1α25(OH)_2_D, whose linear range was 10 to 1000 pg/mL. The LLOQs for all the analytes were 0.5 ng/mL except for 1α25(OH)_2_D, for which the LLOQ was 10 pg/mL. The LOD was 0.29 ng/mL for all the analytes except 1α25(OH)_2_D, for which it was 6 pg/mL. The 7αC4 interference was also cleanly separated from 25OHD3 and 3-epi-25OHD3 [30]. The intraday and interday precision and accuracy values along with the standard deviations are provided in Table 1.

Blank serum was used to evaluate the specificity of our method, and it was found that there were no interfering peaks at the retention times for the compounds analyzed. The absolute recoveries of all the analytes ranged from 75 to 95%. Stability tests were also conducted over three freeze/thaw cycles at 24, 48, and 72 h (hour), and the results were compared with the concentrations at time zero. A 50% deterioration of vitamin D3 was noted at 24 h (time point) after the first freeze/thaw cycle. At 48 h and 72 h, the changes in vitamin D3 concentration were around 15%. For vitamin D2, a 40% change was observed after 24 h, while a 20% change was noted for vitamin D2 during 48 and 72 h. For 25OHD3, the % change in concentration after 24 h was about 40%, while for 48 and 72 h, the changes remained within 15%. 3-epi-25OHD fluctuated between 15% and 60% during the three respective freeze/thaw cycles. A 15% change was noted for the remaining metabolites. This shows that the vitamin D metabolites are not very stable when exposed to continuous freeze–thaw cycles.

**Figure 2:**
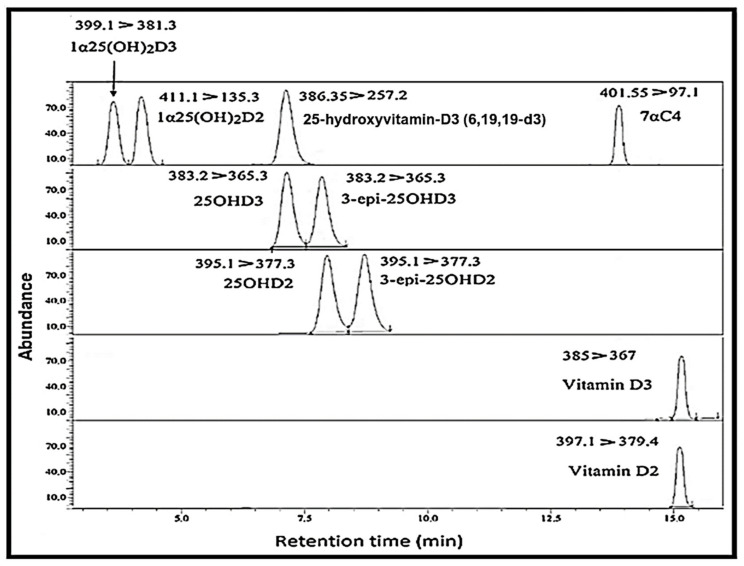
Chromatogram representing the separation of vitamin D metabolites with their respective retention times; [spiked concentrations of 25 ng/mL for all analytes except 1α25(OH)_2_D which were 100 pg/mL].

The chromatographic separation of all the metabolites, along with their retention times and multiple reaction monitoring (MRM) results in the mouse serum, is shown in Figure 2.

**Figure 3:**
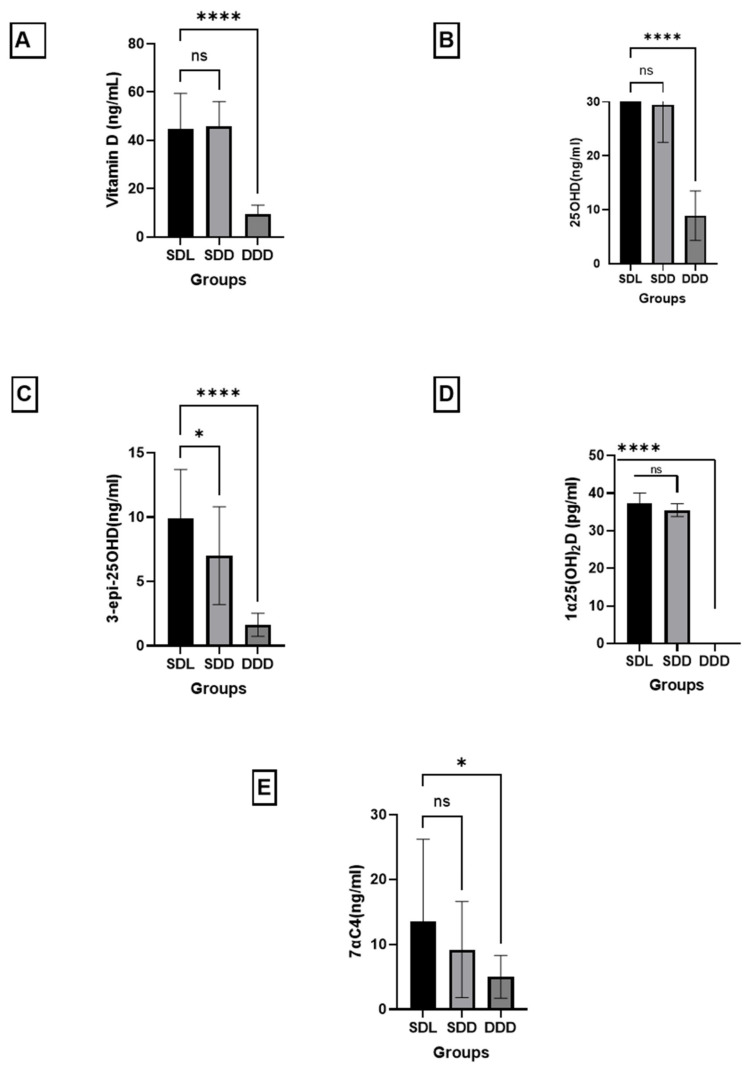
The levels of vitamin D metabolites and epimers in mouse serum, namely, (**A**) vitamin D, (**B**) 25OHD, (**C**) 3-epi-25OHD, (**D**) 1α25(OH)_2_D and (**E**) 7αC4 (the interference, bile acid precursor). Asterisks (* & ****) represent significant differences between group SDL, SDD and DDD. The error bars represent standard deviations. A one-way analysis of variance (ANOVA) (Bartlett’s test) or Student’s *t*-test (two-tailed) was used when required. The differences between the results were considered significant if *p* < 0.05, refuting the null hypothesis (*p* < 0.05). “ns” means no significant difference.

In this study, we report the quantification of vitamin D metabolites in the three groups of mice, fed a vitamin D-deficient diet and kept in the dark (DDD), fed a standard-vitamin D diet and kept in the dark (SDD) and exposed to light along with standard vitamin D diet (SDL). The mouse sample results are represented in Figure 3 as follows.

Figure 3 reveals that there was a significant difference between the SDL group and DDD group of mice for (A) vitamin D, (B) 25OHD, (C) 3-epi-25OHD, (D) 1α25(OH)_2_D and (E)7αC4. It is also clear from the figure that there is a significant difference in the (C) 3-epi-25OHD3 concentrations, and it shows a 30% increase, between the SDD and SDL groups. No significant differences in the concentrations of (A) vitamin D, (B) 25OHD, (D) 1α25(OH)_2_D, and (E) 7αC4 were noted between SDL and SDD groups. Further interpretation of the results shows that the levels of vitamin D were 9.5 ± 3.7, 45.8 ± 10.2, and 44.8 ± 14.6 (mean ± SD) ng/mL in DDD, SDD, and SDL, respectively. The levels of 25OHD were 8.9 ± 4.6, 29.5 ± 7, and 33.1 ± 4.2 ng/mL in DDD, SDD, and SDL, respectively. The C3 epimer, 3-epi-25OHD, also showed an increase to 1.7 ± 0.9, 7.0 ± 3.8, and 9.9 ± 3.8 ng/mL in DDD, SDD, and SDL, respectively.

## 3. Discussion

The serum concentrations of all the metabolites markedly differed between the SDL and DDD groups as shown in Figure 3. The variation in diet and light significantly affected the concentrations of vitamin D metabolites in the three groups of mice. The serum concentrations of all the metabolites markedly differed between the SDL and DDD groups. This may suggest that diet and light both play important roles in increasing and maintaining vitamin D’s metabolites and epimer levels in the blood. The concentrations of all the vitamin D metabolites are reported in the units of ng/mL, except for 1α25(OH)_2_D, which is reported in pg/mL.

On the other hand, the SDL and SDD groups significantly differed (30%) in the concentrations of 3-epi-25OHD metabolites (associated with UV light): This difference is clearly because of the UV light causing an increase in the serum concentrations of the epimers of the vitamin D. These data also suggest that either lower amounts of C3-epimer were produced in the absence of light in the group SDD, or C3-epimers are less efficiently metabolized when vitamin D originates from dietary sources (in the absence of light), such as in the group SDD. SDL group with standard diet and light has caused an increase in the concentration of the C3-epimer. This suggests that the presence of UV light causes an increase in the concentration of epimer in mice serum. This should be further investigated if sun light will have the same effect as the UV light and whether the same phenomenon could happen in humans.

A very similar trend was observed for the 1α25(OH)_2_D concentrations, which were “not detected (ND)”, 35.5 ± 1.7, and 37.7 ± 2.7 pg/mL, in DDD, SDD, and SDL, respectively. This shows that a standard vitamin D intake and UV light increased the levels of all vitamin D metabolites.

Furthermore, the vitamin D, 25OHD and its epimer levels in serum within the group DDD were very low, clearly due to a vitamin D-deficient diet and absence of light. The vitamin D and 25OHD levels were approximately four times higher in SDD than DDD. It was also noted that SDL also showed vitamin D, 25OHD and its epimer concentrations, which were about four times higher than those in the DDD group. Similar trend was observed in 1α25(OH)_2_D in the groups DDD, SDD, and SDL. It was also concluded that, with an increase in vitamin D in the diet and exposure to UV light, the formation of C3 epimers is accelerated. The results also show that vitamin D metabolites acquired from the diet alone are not physiologically equivalent to those obtained by a combination of diet and exposure to UV light. It means that vitamin D rich food, along with light exposure on the skin, can maintain healthy levels of most of the vitamin D metabolites in mice blood. In contrast, only a vitamin D-rich diet in the absence of light is insufficient to maintain optimum levels of vitamin D metabolites in mice blood.

Many animal models have been published in the past to study the effects of vitamin D metabolites and their role in health and disease [38,39,40,41,42,43]. Our study is in agreement with other published animal studies showing that, after vitamin D supplementation or the exposure of the shaved skin of mice to UV radiation, the circulating 25OHD, 1α25(OH)_2_D, and C3 epimer concentrations markedly increase [25]. In other studies, the level of the epimer and its mechanism of exposure to UV radiation were explored, but there were no conclusive results; some hypotheses suggest that the involvement of unknown hydroxylases might be the reason [42,44,45,46]. The cause of vitamin D epimerization and why it occurs differently in different cell types remain unknown, requiring further investigation [20]. An alternative pathway for vitamin D metabolism is CYP11A1 gene activity, which can produce 22OHD3 and 20,22OHD3; the physiological importance of these products remains unclear, but it is possible that UVB light regulates the activity of CYP11A1 and affects the synthesis of epimers [47]. The effect of light on epimer levels was explored in another study, which showed that the levels of 25OHD and 3-epi-25OHD fall during the winter months and reduced light exposure [34].

Moreover, we know that there are fundamental physiological differences in vitamin D metabolism between mice and humans, [48] and we cannot conclude that the current results are directly applicable in humans and it require further investigation. Furthermore, it is known that humans show diurnality and mostly synthesize vitamin D from both sunlight (UVB) and diet, while mice exhibit nocturnality and mostly rely on the oral intake of vitamin D during the night [49]. Therefore, we cannot assume that sun exposure should be preferred over the dietary and oral supplementation of vitamin D in humans. In mice, we clearly observed that dietary vitamin D along with UV exposure increased the concentrations of vitamin D’s metabolites and especially its C3 epimer.

## 4. Materials and Methods

The following standards and reagents were used in the study.

Vitamin D3, vitamin D2, 25OHD3, 25OHD2, 3-epi-25OHD3, 3-epi-25OHD2, 1α25(OH)_2_D2, 1α25(OH)_2_D3, 7αC4, 25-hydroxyvitamin-D3(6,19,19-d3), water, formic acid, ammonium hydroxide, ethyl acetate, methanol, ammonium formate, and acetonitrile, all of LC-MS grade, were obtained from Sigma Aldrich and supplied by LABCO LLC Dubai, UAE. Isopropanol, dichloromethane, and hexane were provided by Emirates Scientific & Technical Supplies LLC (Dubai, UAE).

### 4.1. Mice Sample Analysis

The Ethics Committee of the United Arab Emirates University (UAEU) approved this study. C57BL/6J mice were hosted in a specific pathogen-free (SPF) environment in the animal facility of the Faculty of Medicine and Health Sciences at UAEU.

After weaning at 3 weeks old, 40 mice were divided into three main groups based on the provision of diet and exposure to light. The first group of mice, DDD, was fed a vitamin D-deficient diet (AIN-93G Growing Rodent Diet with 25 IU VD3/kg of diet, D17053003i, Research Diet) and kept in the dark. The second group of mice, SDD (control group), was given a standard-vitamin D diet (Standard AIN-93G Rodent Diet with 1000 IU VD3, D10012Gi, Research Diet) and kept in the dark. The third experimental group of mice, SDL, was kept on a standard-vitamin D diet and exposed to 12-h cycles of UV light and dark, respectively. DDD group contained 10 mice, SDD group and SDL group contained 15 mice each. The three groups of mice were bred in the above conditions for 3 months. For serum collection, the animals were fasted overnight, and whole blood was collected for serum analysis the next day. The mice hair was not shaved from the skin. The detailed information is given in the Table 2 below.

The UV lamps were supplied by Bio-Medical Scientific Services (BIOMSS) LLC, Abu Dhabi. A Philips Xitanium LED Driver 44 W lamp (made in Hungary) emitting broadband LED, 275–900 nm, was used to irradiate mice and to deliver 2.9 kJ/m^2^ of irradiance onto mice. Lamp was held 25–30 cm above the mice.

Blood samples of approximately 300 µL were collected from the three groups of mice (DDD, SDD, and SDL) in a BD vacutainer (part no. 367957) after anesthetizing the mice using diethyl ether solvent. For serum extraction, the blood samples were centrifuged at 1300× *g* for 15 min at 4 °C and stored at −80 °C.

The mouse serum sample (0.3 mL) was thawed at room temperature for 15 min. It was then vortexed. Next, 50 µL of the working internal standard solution of 25-hydroxyvitamin-D3-(6,19,19-d3) was added to all the calibrants, quality controls, and mouse serum samples except the blank. All the vitamin D calibrants and quality controls were prepared in the blank serum. A liquid–liquid extraction was carried out with a mixture of hexane: ethyl acetate (9:1; *v*/*v*) for the extraction of all the analytes from the serum samples. The mixture was first vortexed for a few seconds and then centrifuged at 1320× *g* for 20 min. After the centrifugation, the supernatant’s transparent organic layer was separated and transferred to separate test tubes using Pasteur pipettes. The remaining lower layer was further extracted three times by using the same extraction procedure as described above. All the extracts were pooled together and dried under N2 gas in a sample concentrator. The dried residue was reconstituted in a mixture of 100 µL LC-MS/MS-grade methanol and water (75:25, *v/v*).

We have tried other solvents combinations like acetonitrile and methanol and acetonitrile and water. The best peak shape was obtained with methanol and water (75:25, *v*/*v*). Hexane and ethylacetate was fast evaporating and it was also not recommended for column injection. Ascentis Express F5 column retain vitamin D metabolites by using a stable reversed phase material having electron deficient phenyl rings due to electronegative fluorines in the packing. In addition, the column packing has pi-pi, mildly steric, and mildly polar interactions.

### 4.2. LC-MS/MS System

The LC-MS/MS consisted of a tandem mass spectrometer, model 8060 (Shimadzu, Japan), connected to a Nexera ultra-high-performance liquid chromatography (UHPLC) system (Nexera X2, Shimadzu, Japan). The UHPLC comprises a pump, auto-sampler, column broiler, and degasser. The sensitivity of the analysis was enhanced by using a narrow bore and small-particle-size columns with high pressure tolerance.

The mass spectrometer was used in positive electrospray ionization (ESI) mode. The Shimadzu’s Lab-Solutions software was used for data generation and reporting. The mass spectrometer’s operating parameters for nebulization, drying, heating gas flows and interface, and heating block temperatures were optimized to achieve the best detection and quantification of the vitamin D metabolites. The nebulizing gas flow was set to 2 L/min, drying gas flow was 8 L/min, heating gas flow was optimized at 8 L/min, interface temperature was kept at 300 °C, and heating block temperature was set to 400 °C.

The samples were kept away from direct light to prevent degradation, and the LC-MS/MS sample injection was carried out in dim light. The different metabolites were separated using an Ascentis Express F5 column with dimensions (150 mm × 2.1 mm) and particle size 2.7 µm, fitted with a pre-column guard. To avoid carryover and contamination, the injector needle was rinsed in a mixture of methanol and water (50:50; *v/v*), internally in the UHPLC part before injection into the mass spectrometer. Mobile phase A consisted of aqueous 5 mM ammonium formate, while mobile phase B consisted of methanol with 5 mM ammonium formate. The flow rate was kept at 0.5 mL/min using a binary gradient pump. To achieve optimum separation, a gradient flow was maintained as follows: the initial flow of the mobile phase was 25% A and 75% B (from 0 to 11 min) and then changed to 100% of mobile phase B (from 11 to 15 min) and kept at 100% of mobile phase B (from 15 to 16 min); finally, it was re-equilibrated to 25% A and 75% B (16.1→20 min).

An electrospray ionization (ESI) source was used for ionization and protonation to a molecular ion [M + H]^+^. To achieve the maximum sensitivity, the optimum MRM parameters were fixed for an individual analyte by the direct infusion of individual analytes of the standard compound. The Shimadzu 8060 optimizer software was used for the analyte optimization. The most sensitive MRM transition with the most sensitive precursor and product ions were selected for LC-MS/MS analysis. Most precursor ions were observed after the initial loss of water molecules from vitamin D metabolites. Through chromatographic separation, LC-MS/MS could distinguish between 25OHD3, 3-epi-25OHD3, and 7αC4, even though they had the same ionization precursor and product ions, and the same collision energies. Similarly, 25OHD2 and 3-epi-25OHD2 could also be differentiated based on retention times as having the same precursor ion, product ion, and collision energies. The selected MRM transitions, along with their respective parameters, are given in Table 3.

We monitored 3 MRM transitions (one quantifier and two qualifier ions) for each analyte on a Shimadzu 8060 mass spectrometer by scheduled MRM with a target cycle time of 0.5 s, a retention-time window of 90 s, and an interscan delay of 3 ms. Only one most prominent and high abundance transition was chosen as a quantifier ion, while two less abundant ions were used as qualifier ions. The details of the quantifier and qualifier ions are shown in Table 3 for clarity. Transitions marked with an asterisk are used for quantitation.

### 4.3. Method Validation

The US Food and Drug Administration (FDA) guidelines for bioanalytical method validation were followed to confirm the validity of the method in terms of linearity, specificity, stability, recovery, intraday/interday precision, and accuracy [50]. To evaluate the above parameters, quality controls (QCs), namely, quality control low (QCL = 12 ng/mL), quality control medium (QCM = 50 ng/mL), and quality control high (QCH = 100 ng/mL) were prepared, except for 1α25(OH)_2_D, the QCs for which were prepared at lower concentrations: QCH = 100 pg/mL, QCM = 50 pg/mL and QCL = 12 pg/mL, respectively. The integrity of a validation run was assured by running six QCs at each concentration level (QCL, QCM and QCH) along with a fresh calibration curve each time.

(1) $$\left[\%\;CV=\left(\frac{Standard\;deviation}{mean}\right)\times 100\right]$$

The assay % inter/intra-day accuracy was obtained from the quality control data using the equation:

(2) $$\left[\%\;Accuracy=\left(\frac{Mean\;value}{Nominal\;value}\right)\times 100\right]$$

The % absolute recoveries were calculated using the equation:

(3) $$\left[\%\;Recovery=\left(\frac{Mean\;unextracted\;QC\;values}{Mean\;extracted\;QC\;values}\right)\times 100\right]$$

The lower limit of detection (LOD) was determined by comparing the signal/noise (S/N) ratio with the analyte’s lowest concentration, determined by decreasing the analyte concentration until a response equivalent to three times the background level was observed. Blank serum was used for the preparation of the calibration curve and the quality controls (QCs). Blank serum was prepared by mixing albumin serum with phosphate-buffered saline at a concentration of 60 g/L [51]. For the recovery experiment, six quality control samples at three different levels (QCL, QCM, and QCH) were spiked with the required concentrations of vitamin D metabolites in methanol, and the absolute recovery was calculated. The same experiment was repeated with blank serum samples spiked with quality controls at three similar concentrations followed by an extraction procedure, and then, the recovery was calculated and compared using the peak area results (or the area under the normal curve). A stability experiment was performed by analyzing six quality control samples at three concentration levels (as shown above), carrying out three freeze/thaw cycles of the spiked QCs at 0, 24, 48, and 72 h.

Matrix factor was calculated as the ratio of the peak area of a vitamin D metabolite in the presence of serum matrix (where the analyte was added to the serum matrix and then extracted from matrix) to its peak area in the absence of the matrix (analyte neat solution without any serum matrix present). The normalized percentage matrix factor was calculated as the ratio of the matrix factor of a vitamin D analyte to the matrix factor of the internal standard multiplied by 100. Six different lots of blank serum samples were analyzed in triplicate using electrospray-mass spectrometry. The relative standard deviation did not exceed the 15% range setup by FDA. The normalized percentage matrix effect was between 7 and 14.8% and was demonstrated to be consistent at all concentrations levels.

However, to assess the matrix effects, the mice serum samples were analyzed to evaluate the levels of endogenously present vitamin D metabolites.

No carry-over effect was observed, when wash solution (methanol: water; 50:50; *v*/*v*) was injected following the injection of the extracted serum sample at the highest spiked concentration level. The wash solution chromatogram showed no interfering or coeluting peaks at the retention times of vitamin D metabolites and internal standard. Six different extracted serum samples at highest concentration levels were analyzed in triplicate using electrospray-mass spectrometry to evaluate carry-over effects.

GraphPad Prism version 9.1.2 was used for data analysis. A single factor or one-way analysis of variance (ANOVA) (Bartlett’s test) or Student’s *t*-test (two-tailed test) was used as required. The three groups, DDD, SDD, and SDL were compared to determine if there were any significant differences in the levels of the vitamin D metabolites among the three groups, and the results were considered significant if *p* < 0.05. The significant differences between the groups are represented by asterisks.

## 5. Conclusions

A simple and highly sensitive LC-MS/MS assay was successfully developed and validated for the measurement of vitamin D metabolites, including vitamin D’s epimers, along with a bile acid precursor, 7αC4 (interference) in mouse serum. This method is an improvement on reported assays for the analysis of vitamin D metabolites in biological matrices, which suffer from the need for extensive sample pretreatment and purification, low precision, and metabolite interferences. This method is clean from interference caused by 7αC4 in an LC-MS/MS analysis. The validated method was applied to mouse serum samples for analysis of vitamin D metabolites. The administration of a standard-vitamin D diet increased the concentrations of vitamin D metabolites and its epimer in the mouse serum, and when combined with UV irradiation, it further increased the C3 epimer levels and showed a 30% increase. This study has identified differences in vitamin D metabolites and epimer levels in mice depending on the source of vitamin D.

The increased C3 epimer synthesis with the standard-vitamin D diet and UV exposure could be due to UV light playing a significant part in either the epimer synthesis or limiting its metabolism. There are still some unanswered questions like, Is the UV light equivalent to sunlight exposure? Would we see the same increased epimer levels in humans under UV exposure? Further research is needed to understand the mechanism of action and the reasons for this variability in C3 epimer levels of vitamin D, the role of C3 epimers in human health and disease, and the biochemistry of the C3 epimers and the pathways involved in their generation.

## Figures and Tables

**Table 1:** Intraday–interday precision and accuracy of vitamin D metabolites and 7αC4.

Time	Quality Control Concs.	25OHD3 Conc. (ng/mL)			25OHD2 Conc. (ng/mL)			3-epi-25OHD3 Conc. (ng/mL)			3-epi-25OHD2 Conc. (ng/mL)			7αC4 Conc. (ng/mL)		
		Precision (% CV)	Accuracy %	SD	Precision (% CV)	Accuracy %	SD	Precision (% CV)	Accuracy %	SD	Precision (% CV)	Accuracy %	SD	Precision (% CV)	Accuracy %	SD
Intra-day	QCH 100	6.1	100	6.2	11.7	99	11.2	5.2	114	6.1	5.3	97	8.2	9.3	95	9.2
	QCM 50	9.4	114	7.5	6.6	111	10.1	8.3	106	5.0	10.2	110	6.0	11.1	99	6.4
	QCL 12	7.3	103	1.6	6.4	93	1.4	8.1	108	1.2	4.8	104	6.3	13.6	85	1.1
		1α25(OH)_2_-D3 Conc. (pg/mL)			1α25(OH)_2_-D2 Conc. (pg/mL)			Vitamin-D3 Conc. (ng/mL)			Vitamin-D2 Conc. (ng/mL)					
	QCH 100	5.2	104	5.2	8.4	104	8.5	10.5	103	12.1	13.2	108	4.0			
	QCM 50	14.1	105	7.1	9.7	111	5.4	9.2	101	4.3	11.2	108	6.7			
	QCL 12	8.5	88	1.7	8.8	90	1.9	12.1	85	1.1	16.0	98	12.2			
Inter-day		25OHD3 Conc. (ng/mL)			25OHD2 Conc. (ng/mL)			3-epi-25OHD3 Conc. (ng/mL)			3-epi-25OHD2 Conc. (ng/mL)			7αC4 Conc. (ng/mL)		
	QCH 100	7.5	98	11.3	9.1	95	9.2	8.3	95	7.2	8.0	111	9.1	4.0	96	5.3
	QCM 50	8.3	97	13.1	13.8	114	8.6	7.2	97	16.1	13.7	109	9.5	14.2	100	10.1
	QCL 12	8.1	102	1.6	5.5	96	1.9	6.5	99	1.2	11.1	90	1.5	9.1	75	1.5
		1α25(OH)_2_-D3 Conc. (pg/mL)			1α25(OH)_2_-D2 Conc. (pg/mL)			Vitamin-D3 Conc. (ng/mL)			Vitamin-D2 Conc. (ng/mL)					
	QCH 100	4.5	102	10.2	5.5	99	5.2	6.5	95	6.1	15.2	110	12.3			
	QCM 50	14.8	111	8.3	10.1	109	6.3	14.1	91	7.5	6.0	115	4.0			
	QCL 12	7.0	111	1.9	11.8	96	1.6	8.7	70	2.8	13.5	95	4.7			

**Table 2:** Vitamin D diet and UV light conditions for the three mice groups.

Group	Light(h/day)	Dark (h/Day)	Vitamin D Amount in Diet
SDL	12 h/day	12 h/day	Standard
SDD	0 h/day	24 h/day	Standard
DDD	0 h/day	24 h/day	Deficient

**Table 3:** Vitamin D and its metabolites are shown along with respective precursor/product ions, retention times, and collision energy. Transitions marked with asterisks (*) were used for quantitation.

No.	Analytes	Retention Time (min)	Precursor (m/z)	Product (m/z)	Collision Energy (eV)
1	Vitamin-D3	15.104	385	367 * (quantifier)	−13
				259 (qualifier)	−16
				91	−56
2	Vitamin-D2	15.072	397.1	379.4 *	−17
				69	−19
3	25OHD3	6.98	383.2	365.3 *	−15
				107.1	−30
4	25OHD2	7.76	395.1	377.3 *	−17
				81.1	−38
5	3-epi-25OHD3	7.69	383.2	365.3 *	−15
				107.1	−30
6	3-epi-25OHD2	8.52	395.1	377.1 *	−17
				81.1	−38
7	1α25(OH)_2_D3	3.82	399.1	381.3 *	−14
8	1α25(OH)_2_D2	3.948	411.1	135.3 *	−13
				133.1	−12
9	7αC4	14.48	401.5	383.25	−16
				97.1 *	−29
				91.2	−23
10	IS [25 hydroxyvitamin-D3 (6,19,19-d3)]	6.97	386.3	368.2	−15
				257.2 *	−183
				95.2	−35

## Data Availability

All relevant data are included in the paper.
